# Age distributions in Paralympic Games Paris 2024: an analysis of 5,540 para athletes across 13 individual disciplines

**DOI:** 10.3389/fspor.2025.1720957

**Published:** 2026-01-22

**Authors:** Rafael Lima Kons, Gennaro Apollaro, Rachel Bevins, José Morales, Vinicius de Oliveira Gulias, Sidney Grosprêtre, Kevin De Pauw, Bart Roelands

**Affiliations:** 1 Department of Physical Education, Federal University of Bahia, Bahia, Brazil; 2 Human Physiology and Sports Physiotherapy Research Group (MFYS), Vrije Universiteit Brussel, Brussels, Belgium; 3 Department of Neuroscience, Rehabilitation, Ophthalmology, Genetics and Maternal Child Health, University of Genoa, Genoa, Italy; 4 Sport and Health, Birmingham Newman University, Birmingham, United Kingdom; 5 FPCEE Blanquerna, Universidad Ramon Llull, Barcelona, Spain; 6 Laboratory Culture Sport Health and Society (C3S-UR 4660), Sport and Performance Department, University of Franche-Comte, Besançon, France; 7 Brussels Human Robotic Research Center (BruBotics), Vrije Universiteit Brussel, Brussels, Belgium; 8 Laboratory of Sports and Nutrition Research, Riga Stradiņš University, Riga, Latvia

**Keywords:** competitive achievement, elite Para athletes, long-term Para athlete development, sport-specific age patterns, talent identification

## Abstract

This study aimed to quantify the age distributions of Para athletes competing in the Paris 2024 Paralympic Games across 13 individual disciplines. A total of 5,540 athletes (52.2% male, 47.8% female) participated, with ages calculated at competition. The analysis examined differences by discipline, sex, and competitive achievement (medallist vs. non-medallist). The statistical analyses included the Kruskal–Wallis test followed by Mann–Whitney *U post-hoc* tests to detect differences across disciplines, while Mann–Whitney *U* and chi-square tests were used to analyze sex and competitive achievement at the level of 5%. The results revealed significant variation in age across sports. Para Equestrian (38 years), Para Archery (37 years), and Para Powerlifting (36 years) exhibited the highest ages, indicating later career, while Para Swimming (25 years), Para Taekwondo (27 years), and Para Athletics (29 years) showed earlier ages. The male Para Athletes demonstrated a slightly higher median age than females (30 vs. 28ys). No significant age difference was found between medallists and non-medallists. Age category analysis highlighted a greater proportion of athletes over 30 years in disciplines with later ages. These findings underscore the importance of sport-specific age considerations in Paralympic athlete's development and talent identification. The study contributes novel insights by encompassing multiple disciplines and Para athlete profiles, offering a comprehensive understanding of age-related performance patterns in elite Para sport.

## Introduction

The age at which athletes reach performance is a subject of significant interest within the sports science literature (1, 2). Allen et al. (3) emphasized its importance, while at the same time considering various factors, including sport-specific disciplines, sex, and sports event type. Investigations related to this topic typically focus on three main approaches: identifying the age at which elite athletes achieve their best performances; calculating the average age of top-ranked athletes competing in high level events, such as the Olympic/Paralympic Games; or modelling the trajectory of performance using age-related career data (3–11).

The Paralympic Games have become a global phenomenon, continuously growing in both visibility and participation across a diverse range of disciplines (12). This includes team-based events such as sitting volleyball and five-a-side football, as well as individual disciplines like Para athletics and Para swimming, each contributing to the spectacle and competitive spirit of the Paralympic Games (13). In the Paralympic scenario, especially for individual disciplines, the aspect related to performance in high-level competitions becomes even more complex, involving a series of factors that must be considered in the context of competitions (14, 15). These aspects including the phases of Para sport development pathways (14), classification systems (16), training histories (17), type and origin of impairment (15, 18), as well as age, which, similar to the Olympic context (4), remains a critical factor in the Paralympic scenario (19).

The importance of quantifying the age of performance across different sport disciplines is highlighted by Longo et al. (4), who explored this variable using data from the 2012 London Summer Olympics (*n* = 3548). The authors highlighted that the mean age of female and male athletes was 26 and 27 years, respectively, with 72% of all Olympic athletes falling within the 20–30-year age range. In males, sports such as combat, gymnastics, and swimming were predominantly represented by younger athletes, a trend also observed among females. However, it is important to note that the analysis was based on specific athlete subgroups (*e.g.,* top 20 finishers or medallists), which may limit the generalizability of the findings. Despite the limitations, it is clear that understanding these differences is necessary (19), especially from the perspective of understanding the development aspects of each discipline (19, 20). This is particularly significant in Paralympic sport, where athletes tend to be older than their non-disabled counterparts (15). For instance, in judo, Paralympic athletes are approximately four years older than those competing in the Olympic Games (21).

Based on this perspective, the present study aimed to quantify the age distributions of elite Para athletes competing across 13 individual disciplines at the Paris 2024 Paralympic Games: Para Athletics, Para Swimming, Para Cycling, Wheelchair Tennis, Wheelchair Fencing, Para Powerlifting, Para Judo, Para Triathlon, Para Archery, Para Taekwondo, Para Badminton, Para Equestrian, and Para Rowing. The analysis considered variations in age according to sporting discipline, sex (male and female), and competitive achievement (medallist and non-medallist). This study adopts an exploratory approach across a range of Para athlete profiles to address a gap in the literature by providing a broader and more integrated understanding of age-related performance patterns in Paralympic competition. In this context, examining age distributions as a key indicator, the study also contributes to profiling each discipline within the broader framework of Para sport development pathways. Given the diversity of athlete profiles and developmental trajectories across Para sports, the findings aim to inform evidence-based decision-making in athlete identification, training, and long-term development.

## Methods

### Study design

This descriptive exploratory cross-sectional study examined data on age-related performance among Para athletes competing at the Paris 2024 Paralympic Games from 13 individual sports.

### Participants

A total of 5,540 Paralympic athletes participated in this study, encompassing a broad representation across 13 competitive disciplines. The distribution by Para sport was as follows: Para Athletics had the highest representation with 1,743 athletes, followed by Para Swimming (1,672 athletes), and Para Cycling (658 athletes). Other Paralympic sports included Wheelchair Tennis (*n* = 276), Wheelchair Fencing (*n* = 205), Para Powerlifting (*n* = 179), Para Judo (*n* = 146), Para Triathlon (*n* = 144), Para Archery (*n* = 137), Para Taekwondo (*n* = 121), Para Badminton (*n* = 120), Para Equestrian (*n* = 116), and Para Rowing (*n* = 23). The sample included athletes with intellectual, visual and physical impairments, according to the eligible impairments for the individual sports. The sample included 2,894 male athletes (52.2%) and 2,646 female athletes (47.8%), indicating a relatively balanced participation rate across sexes in elite Para sport. In terms of competitive achievement, 1,478 athletes (26.7%) were classified as medallists, having secured at least one medal in Paralympic Games 2024 (gold, silver or bronze), while 4,062 athletes (73.3%) were non-medallists.

### Data collection and procedures

The Official Results Books contain information on each athlete's name, sex, birth date, competitive achievement (e.g., final classification related to medals or position). Archived databases from open-access websites have been used in similar investigations, and there are no ethical issues involved in the analysis and interpretation of the data, as they were obtained secondarily rather than generated experimentally (22). This approach to data utilization aligns with the ethical principles outlined in the Belmont Report (23), ensuring that participants’ autonomy and confidentiality are upheld. The analysis included multiple factors: Paralympic disciplines (Para Athletics, Para Swimming, Para Cycling, Wheelchair Tennis, Wheelchair Fencing, Para Powerlifting, Para Judo, Para Triathlon, Para Archery, Para Taekwondo, Para Badminton, Para Equestrian, and Para Rowing), sex (male and female), and competitive achievement (medallists and non-medallists). In addition, age distributions were categorized into four groups, based on prior research in Paralympic sport (8–11): under 20 years, 20–25 years, 25–30 years, and over 30 years. The age was calculated using the formula: (competition date—birth date)/365.25. The official start date of the Paris 2024 Paralympic Games (i.e., the opening ceremony) was used as the reference date for all calculations.

### Statistical analysis

The Kolmogorov–Smirnov test revealed the nonnormal distribution of all the considered variables. Therefore, descriptive data are presented as median (interquartile range) [minimum–maximum]. First, the Kruskal–Wallis test followed by the Mann–Whitney *U post-hoc* test was used to detect differences in athletes’ age of different Paralympic disciplines (i.e., Para Archery, Para Athletics, Para Badminton, Para Cycling, Para Equestrian, Para Judo, Para Powerlifting, Para Rowing, Para Swimming, Wheelchair Tennis, Para Taekwondo, Para Triathlon and Wheelchair Fencing). Considering the multiple comparisons, the alpha level was adjusted (*α* ≤ 0.00064) using a Bonferroni correction to eliminate the risk of an inflated Type I error. Effect size *r* was calculated by the formula: *r* = Z/√N; and the magnitudes of the effect size were interpreted with values of 0.1 = *trivial*, 0.1–0.29 = *small*, 0.30–0.49 = *medium*, and ≥0.50 = *large* (24). Second, the Mann–Whitney *U* test was performed to detect differences in age in relation to sex (i.e., female and male), and competitive achievement (i.e., medallist and non-medallist). For these analyses, the effect size *r* was calculated as indicated above. Finally, chi-square (*χ*^2^) tests of independence were performed to identify the association of the Paralympic discipline, sex, and competitive achievement with the age categories (*i.e*, <20, 20–25, 25–30, >30). For these analyses, effect size was reported using Cramer's *V* and the magnitudes were interpreted with values of 0.06–0.17 = *small*, 0.18–0.29 = *medium*, and ≥0.30 = *large* (25). Statistical significance was defined as *p* < 0.05 and all analyses were performed using IBM SPSS Statistics for Windows, version 25.0 (*IBM Corp., Armonk, NY, USA*).

## Results

Table 1 presents a comparison of age distributions across disciplines, sex, and levels of competitive achievement at the Paris 2024 Paralympic Games. A significant difference was observed between disciplines [*χ*^2^_(12)_ = 1,027.035, *p* < 0.001]. To enhance clarity and avoid excessive detail in the main text, only the most relevant pairwise comparisons are summarized below, based on the magnitude of effect size and disciplinary relevance. The complete set of *post-hoc* comparisons, including U-values, *p*-values, and effect sizes, are provided in Supplementary Table S1.

**Table 1:** Comparison of age distributions [median (interquartile range), minimum–maximum] by discipline, sex, and competitive achievement in the 2024 Paris paralympic games (*n* = 5,540).

Discipline	*n*	Age (years)
Para Archery	137	37.0 (31.0–44.5) [16–66]
Para Athletics	1,743	29.0 (25.0–35.0) [15–68] ^A^
Para Badminton	120	30.0 (26.0–36.8) [17–61] ^A^
Para Cycling	658	34.0 (28.0–40.0) [18–60] ^ABC^
Para Equestrian	116	38.0 (30.3–50.8) [21–69] ^BCD^
Para Judo	146	31.0 (26.0–36.0) [18–58] ^ABDE^
Para Powerlifting	179	36.0 (30.0–41.0) [18–59] ^BCEF^
Para Rowing	23	36.0 (28.0–43.0) [19–50] ^B^
Para Swimming	1,672	25.0 (21.0–30.0) [13–59] ^ABCDEFGH^
Para Taekwondo	121	27.0 (23.0–31.5) [16–46] ^ABCDEFGHI^
Para Triathlon	144	34.0 (29.0–39.0) [16–60] ^ABCEFIJ^
Wheelchair Tennis	276	34.0 (27.0–41.0) [15–70] ^ABCEFIJ^
Wheelchair Fencing	205	35.0 (30.0–40.0) [21–68] ^ABCEFIJ^
Sex		
Male	2,894	30.0 (25.0–37.0) [14–70]
Female	2,646	28.0 (24.0–35.0) [13–69]^a^
Competitive achievement		
Medalist	1,478	29.0 (25.0–35.0) [14–69]
Non-Medalist	4,062	29.0 (24.0–36.0) [13–70]

n, number of athletes; The letters indicate the statistical differences for Paralympic Sports: ^A^ = Para Archery; ^B^ = Para Athletics; ^C^ = Para Badminton; ^D^ = Para Cycling; ^E^ = Para Equestrian; ^F^ = Para Judo; ^G^ = Para Powerlifting; ^H^ = Para Rowing; ^I^ = Para Swimming; ^J^ = Para Taekwondo; ^a^Difference for male group.

Notably, Para Archery athletes showed a significantly higher age compared to those in Para Athletics (*r* = 0.20, small), Para Badminton (*r* = 0.31, medium), Para Judo (*r* = 0.32, medium), Para Swimming (*r* = 0.31, medium), and Para Taekwondo (*r* = 0.53, large). Conversely, Para Athletics athletes had a lower age than athletes in Para Cycling (*r* = 0.23, small), Para Equestrian (*r* = 0.20, small), and Para Powerlifting (*r* = 0.19, small), but were older than those in Para Swimming (*r* = 0.29, small) and Para Taekwondo (*r* = 0.09, trivial). Additional comparisons of interest include younger ages in Para Badminton and Para Swimming compared to several other disciplines, although effect sizes were mostly small to trivial and should be interpreted cautiously.

A significant difference was also observed between sexes, with female athletes reaching better performance at a slightly younger age than males (U = 3,459,553.00; *p* < 0.001; r = 0.08, trivial), but the effect size was trivial, indicating that this difference should not be over-interpreted. No significant difference was found between medallists and non-medallists (U = 2,946,004.00; *p* = 0.289; *r* = 0.01, trivial).

**Figure 1:**
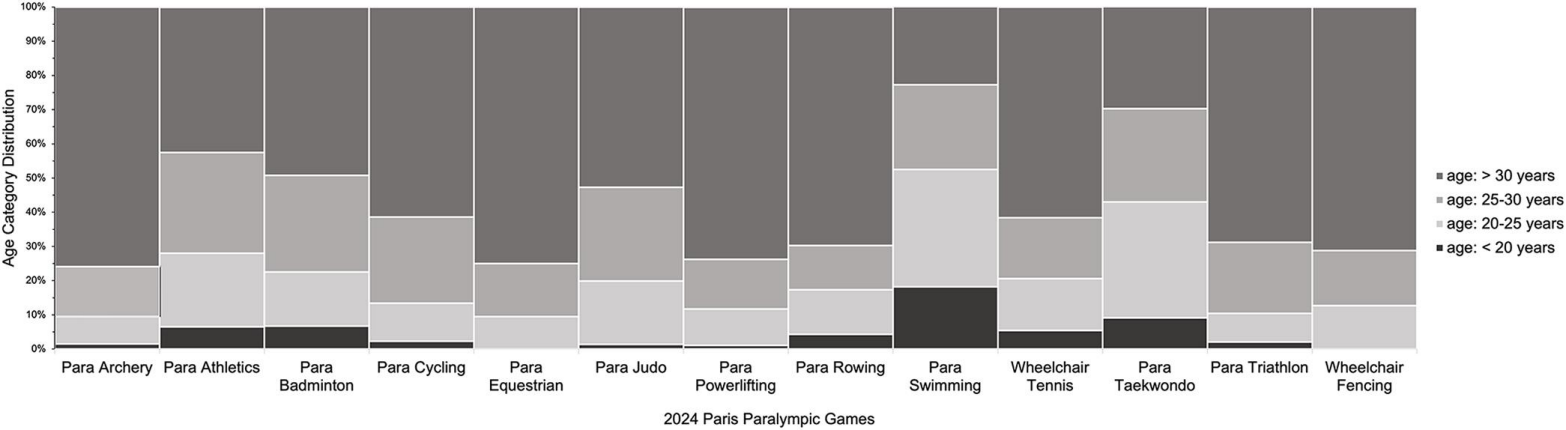
Age category distributions of athletes from different disciplines who participated at the Paris 2024 paralympic games. The stacked bar chart displays the proportion of athletes in four age categories: <20 years, 20–25 years, 25–30 years, and >30 years.

**Figure 2:**
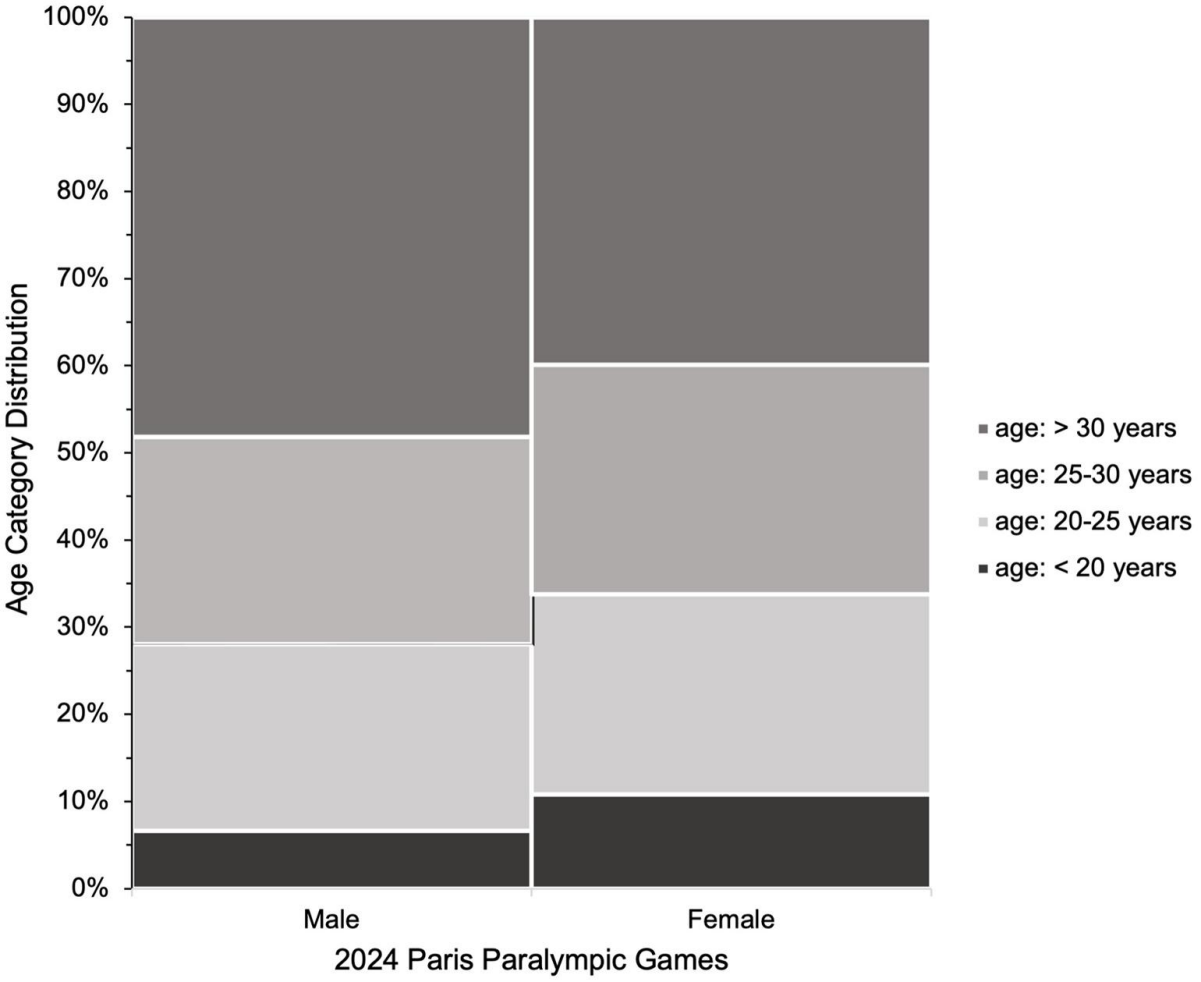
Age category distributions of male and female athletes who participated at the Paris 2024 paralympic games. The stacked bar chart displays the proportion of athletes in four age categories: <20 years, 20–25 years, 25–30 years, and >30 years.

**Figure 3:**
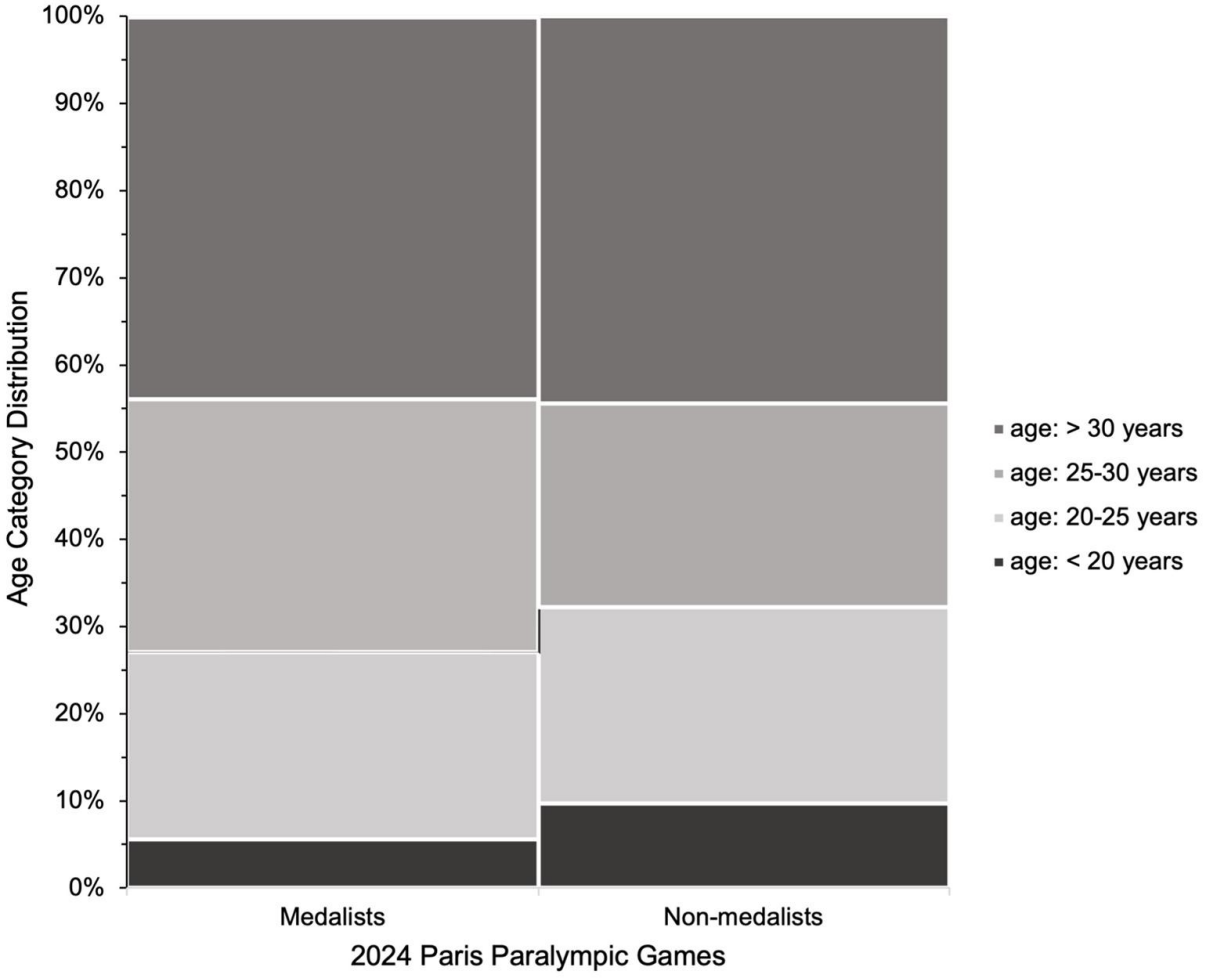
Age category distributions of medallists and non-medallists athletes who participated at the Paris 2024 paralympic games. The stacked bar chart displays the proportion of athletes in four age categories: <20 years, 20–25 years, 25–30 years, and >30 years.

Figures 1–3 present the of age category distributions of the Paralympic disciplines (i.e., Para Archery, Para Athletics, Para Badminton, Para Cycling, Para Equestrian, Para Judo, Para Powerlifting, Para Rowing, Para Swimming, Wheelchair Tennis, Para Taekwondo, Para Triathlon, Wheelchair Fencing), sex (i.e., female and male), and competitive achievement (i.e., medallist and non-medallist) with the age categories distribution (*i.e,* <20, 20–25, 25–30, >30), respectively. Significant associations were found for Paralympic discipline [*χ*^2^_(36)_ = 943.960; *p* < 0.001; *V* = 0.238, medium], sex [*χ*^2^_(3)_ = 54.015; *p* < 0.001; *V* = 0.099, *small*], and competitive achievement (*χ*^2^_(12)_ = 35.111; *p* < 0.001; *V* = 0.080, *small*), indicating a higher distribution of athletes in the group >30 years compared to the other age groups, these findings should be interpreted cautiously given the limited magnitude of the associations.

## Discussion

This study aimed to quantify the age distributions of elite Para athletes competing across 13 individual disciplines at the Paris 2024 Paralympic Games. The main results showed significant variation in age distributions across disciplines, with reach performance later in Para Equestrian, Para Archery, and Para Powerlifting, especially compared to the Para Swimming, and Para Athletics. In the sex analysis, male Para athletes had a slightly higher age than females Para athletes. However, more medallists were over 30 years, especially in Paralympic disciplines with higher ages.

The comparison of the disciplines revealed a significant variation in the age distributions across the 13 Paralympic disciplines (Table 1). Para Equestrian (38 years), Para Archery (37 years), Para Powerlifting (36 years), Wheelchair Fencing (35 years), and Wheelchair Tennis (34 years) were associated with higher age at performance, suggesting that Para athletes in these disciplines tend to reach performance later than other Paralympic disciplines. This trend can be attributed to the nature of these sports, which are predominantly based on precision, control, and technical skill, such as aiming at a target, lifting a load, or effectively communicating with a horse, rather than high-intensity physical exertion. As a result, these disciplines may allow for greater longevity in high-performance careers, as they place relatively lower physical demands on the body compared to more physiologically taxing sports (2, 26, 27).

The lower median ages observed in Para Swimming (25 years), and Para Athletics (29 years) suggest a trend toward earlier specialization in these sports (15). Among them, Para Swimming and Para Athletics stand out as foundational and highly relevant disciplines within the Paralympic system. Both sports have been part of the Paralympic Games since their inception, and are notably inclusive, accommodating athletes with physical, visual, and intellectual impairments (28, 29). Additionally, they stand out for their scale, attracting the highest number of participants among all Paralympic sports, for example, 1,672 in Para Swimming and 1,743 in Para Athletics. The long-standing presence and inclusive nature of these disciplines suggest that their athlete development systems are more mature and diversified (30), providing earlier entry points and sustained development pathways for younger Para Athletes. Furthermore, physical performance is a critical factor in these disciplines, particularly in distance-based events that require high levels of physical conditioning, an area strongly influenced by age (3, 31). In Para Swimming, for instance, age has a significant impact on performance between 12 and 20 years (6). This underscores the critical influence of age on performance development and suggests that in more traditional Paralympics modalities—where physical output is a key factor—athlete development tends to occur earlier. Finally, Para Taekwondo, which has a relatively low performance age of 27 years, is a relatively recent addition having debuted at the Tokyo 2020 Paralympic Games. Its development and classification frameworks are still in the early stages of being established within the academic literature (32).

Descriptive analysis showed that male Para athletes had a slightly higher median age (30 years) compared to females (28 years). A greater proportion of male athletes were over the age of 30, whereas female athletes were more commonly in the 20–30-years age range. Similar age distributions have been reported across various Paralympic sports, including Para Canoeing (5), Para Swimming (6), Wheelchair Racing (7), Wheelchair Rugby (8), Para Powerlifting (9, 10), and Para Judo (11). However, the predominance of male participants in these studies may have influenced the findings, particularly given the small effect sizes observed. In addition, approximately 40% of male and female Para athletes were aged over 30, highlighting a distinct age profile in individual Paralympic sports, one that tends to be older than in Olympic disciplines (4). Other contributing factors may include biological considerations (33), social influence (34) and the specific athlete pool in this edition of the Paralympic Games. Nonetheless, the small effect size suggests that sex may play a relatively minor role compared to sport-specific demands in determining the performance age in elite Para athletes.

There was no significant difference in age distribution between medallists and non-medallists across 13 individual Paralympic disciplines, suggesting that age alone may not be a determining factor in achieving podium success at the elite level. However, the age distribution data (Figure 3) revealed a higher proportion of medallists over the age of 30, particularly in sports where the performance typically occurs later, such as Para Equestrian, Para Powerlifting, and Para Archery. This pattern may reflect the cumulative benefits of experience and long-term skill acquisition (20, 35), as well as the unique characteristics of certain Para sports. These include the influence of weight categories in combat sports, the diversity of impairment types within disciplines like Para Swimming and Para Athletics, and other sport-specific demands. Despite this trend, the absence of statistical significance suggests that other factors, such as training history (17), athlete classification (20), and the nature and and severity of impairment (18), are likely to be more influcential in distinguishing medallists from non-medallists at the Paralympic level.

This study presents several limitations that should be acknowledged. (1); the cross-sectional design, focusing solely on athletes who competed at the Paris 2024 Paralympic Games, captures only a single time point in each athlete's career, (2); There was a notable disparity in the number of participants across disciplines; for instance, Para Athletics and Para Swimming each included over 1,600 Para athletes, while disciplines such as Para Rowing and Para Equestrian had significantly smaller competitors. This uneven distribution may have affected the statistical robustness of comparisons between disciplines. (3); the heterogeneity of impairments and classification categories within each sport complicates the interpretation of age-related performance aspects, as athletes with different functional abilities often follow distinct developmental pathways (15). In addition, as larger and more comprehensive datasets become available, future work would benefit from applying more advanced inferential and predictive modelling approaches (6), such as regression-based frameworks or classification-specific models, to deepen understanding of age-related patterns and performance determinants in Para sport.

## Conclusion

This study identified substantial variation in the age distributions among elite Para athletes across 13 individual disciplines at the Paris 2024 Paralympic Games. Para Sports such as Para Equestrian, Archery, and Powerlifting were associated with later ages (36–38 years), whereas earlier (25–29 years) were observed in disciplines like Para Swimming, Para Taekwondo, and Para Athletics. Male Paralympic athletes were slightly older than their female counterparts, and more medallists were over the age of 30. These findings emphasize the importance of sport-specific and individualized approaches in Para athlete development, talent identification, and long-term performance planning. Tailoring training and support systems to the distinct age-performance trajectories of each Para Sport and athlete will be essential for maximizing potential and sustaining success in elite Paralympic competition.

### Practical applications

Variations in age across different Paralympic disciplines have direct implications for training, athlete development, and support planning. Coaches and multidisciplinary teams should align training programmes with the specific age-performance profiles of each Para sport. For example, sports that favour early performance require earlier talent identification, structured physical development, and proactive injury prevention strategies. In contrast, disciplines with later ages benefit more from long-term technical and tactical development, psychological support (e.g., managing stress and anxiety), and strategies aimed at maintaining performance and extending athletic careers.

## Data Availability

The original contributions presented in the study are included in the article/Supplementary Material, further inquiries can be directed to the corresponding author.
