# Supplementary material for: Age distributions in Paralympic Games Paris 2024: an analysis of 5,540 para athletes across 13 individual disciplines
Source: Front Sports Act Living. 2026 Jan 22;7:1720957. doi: 10.3389/fspor.2025.1720957 (PMC12872764; doi:10.3389/fspor.2025.1720957)
Supplement: Supplementary file 1 [file Table1.docx]

**Supplementary Table 1.** Pairwise comparisons of age at peak performance between Paralympic disciplines.

| **Discipline A** | **Discipline B** | **U Value** | **p Value** | **Effect Size (r)** | **Classification** |
| --- | --- | --- | --- | --- | --- |
| Para Archery | Para Athletics | 66100.00 | < 0.001 | 0.20 | Small |
| Para Archery | Para Badminton | 5264.50 | < 0.001 | 0.31 | Medium |
| Para Archery | Para Cycling | 36698.00 | 0.001 | 0.12 | Small |
| Para Archery | Para Judo | 6318.50 | < 0.001 | 0.32 | Medium |
| Para Archery | Para Swimming | 37244.50 | < 0.001 | 0.31 | Medium |
| Para Archery | Wheelchair Tennis | 15285.00 | 0.002 | 0.16 | Small |
| Para Archery | Para Taekwondo | 3205.50 | < 0.001 | 0.53 | Large |
| Para Archery | Para Triathlon | 7993.00 | 0.006 | 0.16 | Small |
| Para Archery | Wheelchair Fencing | 11817.50 | 0.013 | 0.13 | Small |
| Para Athletics | Para Cycling | 405667.00 | < 0.001 | 0.23 | Small |
| Para Athletics | Para Equestrian | 52579.50 | < 0.001 | 0.20 | Small |
| Para Athletics | Para Swimming | 973979.00 | < 0.001 | 0.29 | Small |
| Para Athletics | Para Taekwondo | 83744.50 | < 0.001 | 0.09 | Trivial |
| Para Badminton | Para Equestrian | 4164.50 | < 0.001 | 0.35 | Medium |
| Para Badminton | Para Swimming | 59977.50 | < 0.001 | 0.17 | Small |
| Para Cycling | Para Taekwondo | 20412.50 | < 0.001 | 0.31 | Medium |
| Para Judo | Para Swimming | 66146.50 | < 0.001 | 0.22 | Small |
| Para Powerlifting | Para Swimming | 53431.50 | < 0.001 | 0.33 | Medium |
| Para Powerlifting | Para Taekwondo | 4627.00 | < 0.001 | 0.49 | Medium |
| Para Swimming | Wheelchair Tennis | 115256.00 | < 0.001 | 0.30 | Medium |
| Para Swimming | Para Triathlon | 48811.00 | < 0.001 | 0.28 | Small |
| Para Taekwondo | Wheelchair Fencing | 5624.00 | < 0.001 | 0.46 | Medium |
